# SPECT Imaging of *P. aeruginosa* Infection in Mice Using ^123^I-BMIPP

**DOI:** 10.3390/pharmaceutics16050656

**Published:** 2024-05-14

**Authors:** Yuri Nishiyama, Asuka Mizutani, Masato Kobayashi, Yuka Muranaka, Kakeru Sato, Hideki Maki, Keiichi Kawai

**Affiliations:** 1 Division of Health Sciences, Graduate School of Medical Sciences, Kanazawa University, 5-11-80 Kodatsuno, Kanazawa 920-0942, Japan; yuri.nishiyama@shionogi.co.jp (Y.N.); sk-swimlove0323@outlook.jp (K.S.); 2 Laboratory for Drug Discovery & Disease Research, Shionogi & Co., Ltd., 3-1-1 Futaba-cho, Toyonaka 561-0825, Japan; hideki.maki@shionogi.co.jp; 3 Faculty of Health Sciences, Institute of Medical, Pharmaceutical and Health Sciences, Kanazawa University, 5-11-80 Kodatsuno, Kanazawa 920-0942, Japan; mizutani.a@staff.kanazawa-u.ac.jp (A.M.); kobayasi@mhs.mp.kanazawa-u.ac.jp (M.K.); 4 Department of Radiological Technology, Faculty of Health Science, Juntendo University, 2-1-1 Hongo, Bunkyo-ku, Tokyo 113-8421, Japan; y.muranaka.vt@juntendo.ac.jp; 5 Biomedical Imaging Research Center, University of Fukui, 23-3 Matsuoka-shimoaizuki, Eiheiji-cho, Yoshida-gun, Fukui 910-1193, Japan

**Keywords:** *Pseudomonas aeruginosa*, SPECT, ^123^I-BMIPP, difficult to treat, noninvasive

## Abstract

*Pseudomonas aeruginosa* infection is an infectious disease that must be controlled because it becomes chronic and difficult to treat, owing to its unique system of toxin production/injection and elimination of other bacteria. Here, we noninvasively monitored *P. aeruginosa* using single-photon emission computed tomography (SPECT) imaging. Determining the amount and localization of the *P. aeruginosa* will enable making faster clinical diagnoses and selecting the most appropriate therapeutic agents and methods. Nonclinically, this information can be used for imaging in combination with biofilms and toxin probes and will be useful for discovering drugs targeting *P. aeruginosa*. To study *P. aeruginosa* accumulation, we conducted in vitro and in vivo studies using iodine-123 β-methyl-*p*-iodophenyl-pentadecanoic acid (^123^I-BMIPP), which we previously reported using for *Escherichia coli*. In vitro, ^123^I-BMIPP accumulated in *P. aeruginosa* by being taken up into the bacteria and adsorbing to the bacterial surface. In vivo, ^123^I-BMIPP accumulated significantly more in infected sites than in noninfected sites and could be quantified by SPECT. These results suggest that ^123^I-BMIPP can be used as a probe for *P. aeruginosa* for SPECT. Establishing a noninvasive monitoring method using SPECT will allow further progress in studying *P. aeruginosa*.

## 1. Introduction

Although many antibacterial and antiviral drugs have been developed and marketed, the threat of infectious diseases continues, and approximately 2.6 million people died in 2020 from three major infectious diseases: human immunodeficiency virus (HIV), tuberculosis, and malaria [1]. Various drugs are available to treat bacterial infections, but many cases are difficult to treat due to the rise of multidrug-resistant bacteria [2,3,4], biofilm production, and emergence of persister states in infected hosts [5,6].

Definitively diagnosing infectious diseases in clinical practice mainly involves detecting bacteria and identifying bacterial species by culturing specimens such as sputum and blood [7]. Because the number of bacteria contained in these specimens varies depending on the location and collection method, the percentage of bacteria that can be detected in these specimens is low [8,9]. Therefore, infectious diseases are diagnosed comprehensively from modalities such as symptoms, blood tests, and X-ray computed tomography (CT). For cancer and brain diseases, imaging methods using nuclear medicine, such as positron emission tomography and single-photon emission computed tomography (SPECT) [10], are frequently used in addition to X-ray CT. These methods provide information on the activity status of organs and cells by using probes that match each purpose and enable determining the accumulation site and amount. Applying these imaging methods for infectious diseases allows for the noninvasive identification of the infection site within the host and counting bacterial numbers in real time.

Here, ^123^I-β-methyl-*p*-iodophenyl-pentadecanoic acid (^123^I-BMIPP), which was used clinically and previously confirmed to accumulate in *Escherichia coli* [11], was used to evaluate *P. aeruginosa*. ^123^I-BMIPP is a probe used to evaluate fatty acid metabolism in the myocardium. ^123^I-BMIPP is labeled by ^123^I on fatty acid side chains and exhibits the same pharmacokinetics as fatty acids in the body [12]. Evaluating ^123^I-BMIPP uptake can reveal local fatty acid metabolic disorders in the myocardium. 

Unlike *E. coli*, which is easily treated with drugs, *P. aeruginosa* infection is a refractory infection that is difficult to control, and once it develops, it often becomes chronic. The causes of refraction include resistance (e.g., multidrug resistance, biofilm, and persisters) [5,6,13,14], host cell attack by toxin production/toxin injection [15], and other bacterial elimination mechanisms by the T6SS system [16]. *P. aeruginosa* is also the main infecting bacterium in cystic fibrosis, a disease that causes refractory infections due to decreased ability of the lungs to remove foreign substances owing to genetic mutations and repeated acute exacerbations. Cystic fibrosis is associated with a high probability of death in adulthood [17].

Using SPECT with ^123^I-BMIPP, which is commercially available as an infectious disease probe, to noninvasively monitor *P. aeruginosa* will reveal bacterial numbers and the organ distribution in *P. aeruginosa* infections, which have high mortality rates. This will allow for the selection of the appropriate therapeutic drugs and administration methods that account for organ distribution and improved prognoses.

We investigated ^123^I-BMIPP accumulation in *P. aeruginosa* under various conditions in vitro and the organ distribution and accumulation at the infection site in a *P. aeruginosa* mouse thigh infection model. The results suggest that ^123^I-BMIPP may be used as a probe for *P. aeruginosa* infection. We also conducted imaging of a *P. aeruginosa* infection model using an animal SPECT device and determined bacterial numbers from the SPECT images.

## 2. Materials and Methods

### 2.1. Microorganisms

*P. aeruginosa* SR24 is the clinical isolate strain. For the in vitro assay, the bacterial numbers were counted via optical density and used. For the in vivo assay, a stock solution of *P. aeruginosa* SR24 stored at −80 °C was used. The bacterial numbers were also determined by plating on Brain-Heart Infusion Agar (Becton, Dickinson and Co., Franklin Lakes, NJ, USA).

### 2.2. Animals

The Institutional Animal Care and Use Committee of Shionogi and Co., Ltd. (Osaka, Japan) approved all animal study procedures. Specific-pathogen-free male ICR mice (CLEA Japan Inc., Tokyo, Japan, 5 weeks old) were used in all in vivo studies.

### 2.3. In Vitro Accumulation of ^123^I-BMIPP to P. aeruginosa SR24

*P. aeruginosa* SR24 was precultured on Todd–Hewitt Broth (THY) medium (Becton, Dickinson and Co.) and 0.2% yeast extract (Becton, Dickinson and Co.). Thereafter, the bacteria were cultured in Dulbecco’s modified Eagle’s medium (D-MEM, Fujifilm Wako Pure Chemical Industries, Ltd., Hiratsuka, Japan) containing no amino acids for 1 h (lag phase), 3 h (log phase), and 6 h (stationary phase). The settings of the three phases were determined by conducting a growth study in advance.

^123^I-BMIPP (Nihon Mediphysics, Tokyo, Japan) prepared at 37 kBq/10 μL was injected into *P. aeruginosa* SR24 cultured for each time period, incubated at 37 °C for 5 min, and centrifuged at 7000× *g* at 4 °C for 10 min. After centrifugation, the supernatant was removed, and phosphate-buffered saline (PBS, pH 7.4, Takara Bio, Kusatsu, Japan) was added to loosen the pellet. Then, the pellet was centrifuged and washed in the same manner as above. After washing again, the supernatant was removed, and 1.0 mL of 0.1 N NaOH aqueous solution (Nacalai Tesque, Kyoto, Japan) was added to dissolve the bacterial cells. Thereafter, the radioactivity accumulation in *P. aeruginosa* SR24 was measured using a gamma counter. The accumulation rate was calculated using the formula: accumulation rate (%ID) = counts of sample (cpm)/counts of injected radioactivity (cpm) × 100. Bacteria at each culture time point were counted using the colony-counting method [18], and the accumulation rate per number of bacteria was calculated. Specific-pathogen-free male ICR mice (CLEA Japan Inc., 5 weeks old) were used all in vivo studies.

### 2.4. Effect of Temperature on Accumulation

To examine the effects of low temperature, *P. aeruginosa* SR24 was cultured at 37 °C for each amount of time, then cooled to 4 °C for 30 min. ^123^I-BMIPP (37 kBq/10 μL) was added to the bacterial culture and incubated at 37 °C for 5 min, and then the accumulation was measured via the same procedure as above. To determine the effects of high temperature, after culturing for each amount of time, the bacterial culture was heated at 80 °C for 30 min, and the same procedure as that at low temperature was performed. As a standard condition, the bacterial culture was also incubated at 37 °C for 30 min, and the results after 1 h of culturing and incubation at 37 °C were taken as 100% for comparison.

### 2.5. Effect of CD36 Inhibitor on Accumulation

Sulfo-*N*-succinimidyl oleate (SSO, Cayman Chemical, Ann Arbor, MI, USA) [19], an inhibitor of CD36, a membrane protein in the fatty acid transport system in human cells, was prepared with distilled water to a final concentration of 1, 10, or 100 μmol/L. Prepared SSO and 37 kBq/10 μL of ^123^I-BMIPP were added at the same time to *P. aeruginosa* SR24 cultured at 37 °C for each time period. After incubation at 37 °C for 5 min, the accumulated radioactivity was measured via the same procedure as above, and the accumulation rate was calculated.

### 2.6. Biodistribution of ^123^I-BMIPP in a P. aeruginosa SR24 Mouse Thigh Infection Model

Mice were anesthetized with isoflurane, and *P. aeruginosa* SR24 at 4.2 × 10^7^ CFU/0.1 mL saline was aseptically injected into the left thigh of each mouse. At 1 h postinfection, 10 kBq/0.2 mL ^123^I-BMIPP was injected via the tail vein. Mice were euthanized after blood sampling under anesthesia, and each organ was excised at 1, 2, and 4 h postinjection. The organs were weighed, and the radioactivity accumulation was counted using a gamma counter. Each organ accumulation was calculated as a percentage of the ID per gram of wet tissue (%ID/organ, Table 1). The probe’s performance was also evaluated by calculating the accumulation contrast between the uninfected (right thigh) and infected (left thigh) sites (Table 2).

### 2.7. SPECT Imaging of ^123^I-BMIPP in a P. aeruginosa Mouse Thigh Infection Model

One hour postinfection using the same procedure as above, 10–20 MBq/0.2 mL ^123^I-BMIPP was injected via the tail vein. ^123^I-BMIPP accumulation in the bacterial infection site in the mice was imaged via SPECT/CT (Triumph II SPECT 2H/XO SRI CT, TriFoil Imaging). At 1, 2, and 4 h after ^123^I-BMIPP injection, mice were anesthetized with isoflurane, then arranged lying face down on a SPECT/CT bed with both hind legs spread out and fixed with surgical tape. SPECT imaging was acquired under the following conditions: energy window, 20% at 140 keV; projection limit, 30 s; projection count, 64; rotation angle, 360 degrees; and collimator, N5F75A10. The total time for actual imaging was ≈40 min. For image processing, adjusted regions of interest were drawn over the entire infected (left) thigh and contralateral (right) thigh. The %ID was calculated by dividing the accumulation by the radioactivity administered. Contrast was calculated using the same method as above.

### 2.8. Statistical Analysis

The differences between accumulations were evaluated using Student’s *t*-test. *p* < 0.05 was considered statistically significant and accepted within 95% confidence limits using SAS^®^ Studio (SAS Institute Inc., Cary, NC, USA). All results are reported as means ± SD.

## 3. Results

### 3.1. In Vitro Accumulation of ^123^I-BMIPP in P. aeruginosa SR24

**Figure 1:**
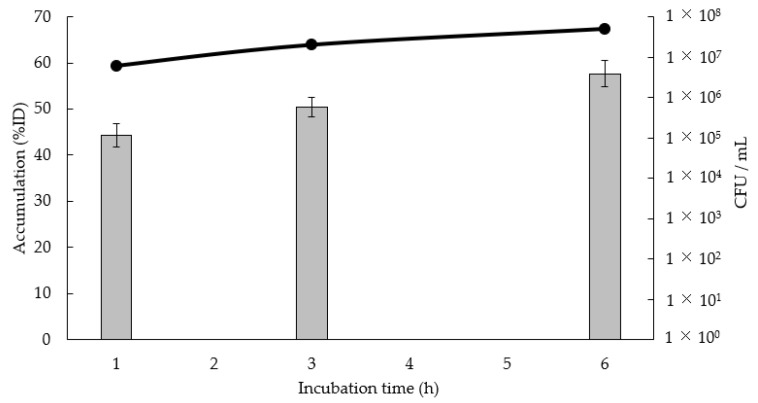
In vitro accumulation of ^123^I-BMIPP for *P. aeruginosa* SR24 (line graph, left axis) and bacterial numbers (bar graph, right axis). Accumulation is expressed as the mean ± standard deviation (SD) for three tests. The bacterial number is expressed as the average of two tests.

Figure 1 shows the ^123^I-BMIPP accumulation in *P. aeruginosa* SR24 at each culture time point. ^123^I-BMIPP accumulation was 44.3% injected dose (ID) at 1 h, 50.4% ID at 3 h, and 57.7% ID at 6 h; thus, the accumulation increased as the culture time increased. The bacterial numbers at each culture time point were 6 × 10^6^ colony-forming units (CFU)/mL at 1 h, 2 × 10^7^ CFU/mL at 3 h, and 5 × 10^7^ CFU/mL at 6 h; thus, the accumulation increased as the bacteria grew.

### 3.2. Effect of Temperature on Accumulation

**Figure 2:**
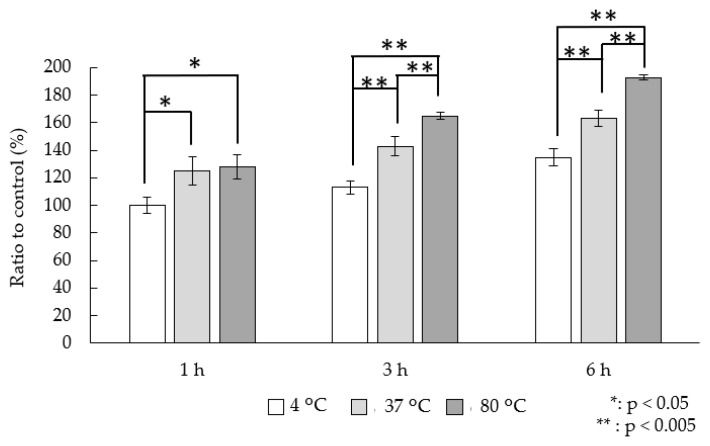
In vitro accumulation ratio relative to the control group at each incubation temperature. Results are expressed as means ± SD for three tests.

We examined the influence of incubation temperature on ^123^I-BMIPP accumulation. Bacteria heated at 80 °C for 30 min did not grow in the culture; thus, the bacteria appeared to be dead. The accumulation rates shown consider that accumulation is 100% at an incubation temperature of 4 °C and an incubation time of 1 h (Figure 2). At 4 °C, 37 °C, and 80 °C, the accumulations were 100%, 125%, and 128% at 1 h; 113%, 143%, and 165% at 3 h; and 135%, 163%, and 193% at 6 h, respectively.

### 3.3. Effect of CD36 Inhibitor on Accumulation

**Figure 3:**
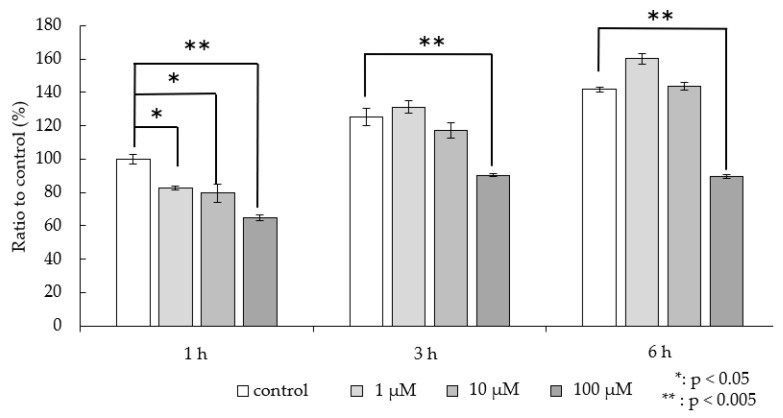
In vitro accumulation ratio relative to the control group accumulation (1 h) in the presence of SSO. Results are expressed as means ± SD for three tests.

Because ^123^I-BMIPP is transported by a fatty acid transport membrane protein in human cells, we investigated the effects of SSO, an inhibitor of the fatty acid transport membrane protein, CD36. Figure 3 shows the accumulation rates after adding SSO. The *P. aeruginosa* accumulation at each time point decreased as the SSO loading concentration increased and was significantly reduced in the 100-µM SSO group compared with that of the control group at all time points.

### 3.4. Biodistribution of ^123^I-BMIPP in P. aeruginosa SR24 Mouse Thigh Infection Model

The biodistribution results (Table 1) showed that nearly 7%ID of the ^123^I-BMIPP remained in the blood after 4 h. For the organ distribution, the highest concentration was in the heart, at approximately 20% after 1 and 2 h and approximately 10% after 4 h. The next highest accumulations were in the kidneys and lungs.

The infection site selectivity of ^123^I-BMIPP was evaluated by calculating the contrast between the accumulation in the uninfected site (right thigh) and the infected site (left thigh; Table 2). The accumulation percentages in the infected site at 1, 2, and 4 h postinfection were 6.3%, 6.7%, and 5.6% ID/g, respectively. The accumulation percentages in the uninfected sites were 4.8%, 4.3%, and 2.8% ID/g, respectively, and the amount of accumulation decreased as the postinjection time increased. The contrast values between the infected and uninfected sites were 1.3, 1.6, and 2.0 at 1, 2, and 4 h, respectively, and accumulation at the infected site was confirmed from 1 h postinfection. The contrast increased as postinjection time increased.

### 3.5. SPECT Imaging of ^123^I-BMIPP in a P. aeruginosa Mouse Thigh Infection Model

**Figure 4:**
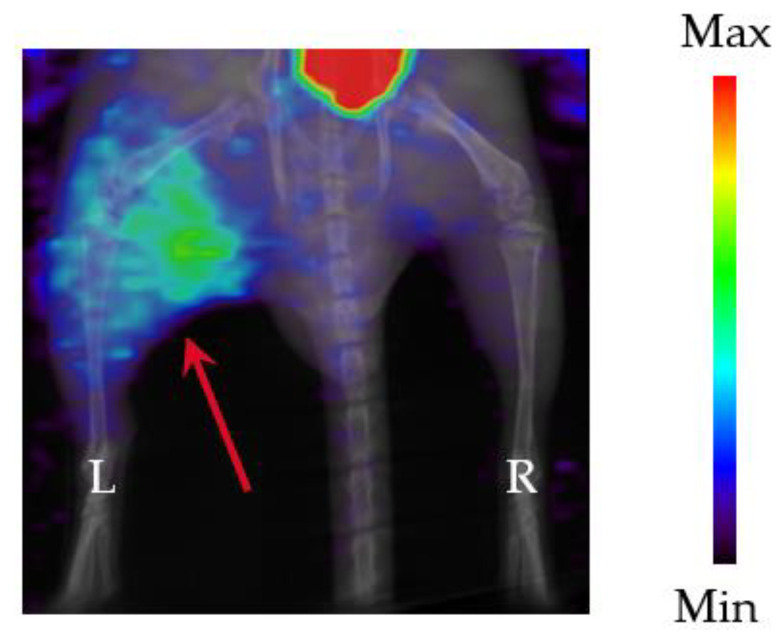
Representative SPECT image (maximum intensity projection (MIP)) of ^123^I-BMIPP in a *P. aeruginosa* SR24 mouse thigh infection model. Mice were infected in the left thigh (red arrow), and images were acquired 4 h after ^123^I-BMIPP administration.

Figure 4 shows a SPECT image of the lower body of a *P. aeruginosa* SR24 thigh infection model mouse. The left thigh (red arrow) is the infection site. The area in the upper part of the figure containing a large amount of accumulation is the bladder, which is the ^123^I-BMIPP excretion site. Although a small amount of ^123^I-BMIPP accumulation was observed at the uninfected site (right thigh), the ^123^I-BMIPP was clearly accumulated at the infected site (left thigh) compared with the uninfected site.

Table 3 shows the contrast values between the accumulation in the uninfected (right thigh) and infected (left thigh) sites calculated from SPECT images. The accumulation percentages at the infected site 1, 2, and 4 h postinfection were 6.0%, 4.9%, and 5.4% ID, respectively. The accumulation percentages in the uninfected sites were 4.0%, 2.9%, and 2.3% ID, respectively; thus, the accumulation percentages decreased as the postinjection time increased. Furthermore, the contrast values between the infected and uninfected sites were 1.5, 1.7, and 2.3 at 1, 2, and 4 h, respectively. These results were similar to those obtained by using the γ counter.

## 4. Discussion

In this study, we evaluated SPECT imaging for *P. aeruginosa*, which is often difficult to treat in clinical practice. *P. aeruginosa* is a resident bacterium in the intestinal tract. When it invades the blood due to external factors, it is difficult to control and can cause bacteremia and sepsis, leading to endotoxin shock due to toxin excretion and death due to multiple organ failure [20]. When treating respiratory infections caused by other causative bacteria, various antibiotics are administered to eliminate antibiotic-sensitive bacteria. Consequently, antibiotic-resistant *P. aeruginosa* has emerged, replacing susceptible bacteria in some cases [21]. Noninvasively determining the amount and distribution of *P. aeruginosa* in the host body using commercially available ^123^I-BMIPP and SPECT will enable speeding up diagnoses and selecting the most appropriate treatment option in clinics. In nonclinical settings, SPECT with ^123^I-BMIPP will be useful for researching therapeutic drugs against *P. aeruginosa* and its toxins and biofilms.

Our results showed that for each culture time point, the ^123^I-BMIPP accumulation in *P. aeruginosa* SR24 increased as the culture time increased. Both the ^123^I-BMIPP accumulation and the bacterial numbers increased as the culture time increased. Thus, ^123^I-BMIPP accumulation may increase in correlation with bacterial numbers.

At varying incubation temperatures, the ^123^I-BMIPP accumulation was lowest at 4 °C, likely because of low uptake resulting from low metabolic activity at this temperature. Because the ^123^I-BMIPP accumulation decreased by approximately 20% at 4 °C compared with that at 37 °C, an uptake or intracellular convection mechanism may have occurred dependent on metabolic activity [22]. Likewise, a certain amount of nonspecific accumulation may have occurred independent of metabolic activity. The difference between 4 °C and 37 °C, which is thought to be dependent on metabolic activity, was always approximately 20%. Conversely, the nonspecific accumulation, as shown by the amount of accumulation at 4 °C, increased as the bacteria grew, likely due to adsorption of ^123^I-BMIPP to the bacterial surface. At 80 °C, which represented accumulation on dead bacteria, the increase was the same (≈20%) compared with that of the control group, suggesting possible specific adhesion to denatured bacterial components. ^123^I-BMIPP accumulation may involve an active mechanism that specifically takes it up at 37 °C and a passive mechanism that takes it up nonspecifically, even at 4 °C, and because it accumulates even after heat treatment, adhesion to dead bacterial components also occurs. In vivo test results suggested that dead bacteria may be eliminated from the infected site via host immunity, and the accumulation in the dead bacteria likely did not affect the imaging [23].

A clinical study reported that all 47 enrolled participants lacking myocardial ^123^I-BMIPP accumulation had genes with type I CD36 deficiency [24]. CD36 is a fatty acid transporter in humans and is present in platelets, monocytes, macrophages, adipose tissue, and skeletal muscle in addition to the myocardium. Because ^123^I-BMIPP is thought to be taken up via CD36, we conducted an experiment using SSO, a CD36 inhibitor. The accumulation rate decreased significantly when high doses of SSO were added, indicating that an uptake mechanism sensitive to SSO is involved in ^123^I-BMIPP accumulation in *P. aeruginosa* SR24, similar to that in human cells. Similar uptake was previously confirmed in *E. coli* [11], indicating a possible common ^123^I-BMIPP uptake mechanism between both species. In future studies, we will investigate whether this ^123^I-BMIPP accumulation is common to other bacterial species.

The biodistribution results indicated that ^123^I-BMIPP accumulated largely in the heart, as it is a probe for myocardial imaging. After ^123^I-BMIPP is intravenously injected, it is taken up into various tissues and metabolized to ^123^I-PIPA. Next, it undergoes glutamine conjugation or glucuronidation in the liver. The high accumulation in the kidneys likely occurred because it is excreted primarily in the urine as a water-soluble substance [25]. The next highest concentration was in the lungs, which have a high blood flow. ^123^I-BMIPP exhibits similar pharmacokinetics to fatty acids, and fatty acids circulate in the blood in the form of albumin or triacylglyceride with lipoproteins [26] (pp. 87–116). Therefore, the concentration in the blood was relatively high.

For the uninfected sites, because the ^123^I-BMIPP accumulation decreased over time, the accumulation was likely nonspecific and decreased as it was metabolized. Accumulation at the infected site was also likely due to the addition of nonspecific accumulation and accumulation in *P. aeruginosa*. Consequently, the contrast became high, and SPECT images confirmed clear accumulation. Furthermore, the contrast results obtained using a quantitative gamma counter were similar to those of the SPECT images, suggesting the possibility of using SPECT image analysis to count bacterial numbers noninvasively.

Compared with previous study results for *E. coli* [11], the in vivo contrast was higher for *P. aeruginosa*, and ^123^I-BMIPP showed higher sensitivity for *P. aeruginosa* than for *E. coli*. Furthermore, using SPECT images enabled visually and easily confirming ^123^I-BMIPP accumulation at the infection site, which is an important element of imaging.

In other non-clinical reports, bioluminescence imaging has been used [27]. Optical imaging is useful for detecting infection sites close to the surface, but it is not suitable for detecting deep infections and cannot be applied clinically. On the other hand, radio isotope-based SPECT imaging allows for imaging and quantification regardless of the depth of the infection site. Alternatively, ELISA has been suggested as a possible biomarker for *P. aeruginosa* by detecting the pseudomonas quinolone signal (PQS) [28] or pyocyanin (PYO) [29] specific to *P. aeruginosa*. However, these immunochemical assays are ex vivo assays using patient samples and therefore cannot evaluate the localization and number of *P. aeruginosa* in the patient’s body. SPECT imaging can non-invasively obtain these data, enabling the selection of suitable therapeutic drugs and administration methods based on biodistribution, ultimately improving prognoses.

These results suggest that ^123^I-BMIPP is useful for detecting *P. aeruginosa*, which is difficult to treat in clinical practice and sometimes causes death. SPECT imaging will allow imaging *P. aeruginosa* localization and evaluating bacterial numbers. Using SPECT to noninvasively image the infection state in an animal’s body by monitoring the same individual over time will allow confirming the growth of infectious bacteria and treatment effects without interindividual differences. Additionally, because culturing is not required to confirm bacterial numbers, the data can be obtained in real time. Biofilms and toxins are factors that make *P. aeruginosa* difficult to treat. Performing imaging and analysis using a combination of probes for these and ^123^I-BMIPP will allow for the quantifying and determining of the distribution of *P. aeruginosa* and the biofilm and toxins it secretes. ^123^I-BMIPP and SPECT will be useful for drug discovery targeting these diseases. Furthermore, ^123^I-BMIPP is a probe used clinically and can be applied to diagnose infectious diseases in clinics.

## 5. Conclusions

^123^I-BMIPP enables detecting *P. aeruginosa*, which is difficult to treat in clinical practice and can cause death. Determining noninvasively bacterial numbers and *P. aeruginosa* localization in real time will help speed up diagnoses in clinics and enable selecting the most appropriate therapeutic agents and methods. Nonclinically, ^123^I-BMIPP can be used for imaging in combination with biofilms and toxin probes through monitoring the same individual animal over time, which will be useful for drug discovery targeting these factors in conjunction with the ethical aspects of laboratory animal testing. Establishing a noninvasive monitoring method using SPECT will enable further study of *P. aeruginosa* infectious disease.

## Figures and Tables

**Table 1:** Biological distribution of ^123^I-BMIPP by organ at 1, 2, and 4 h post-injection in a *P. aeruginosa* SR24 mouse thigh infection model.

	^123^I-BMIPP Accumulation (%ID/g)		
Organ	1 h	2 h	4 h
Heart	21.26 ± 2.35	20.97 ± 2.66	10.18 ± 2.62
Lung	5.91 ± 1.46	4.96 ± 0.95	3.71 ± 0.26
Liver	2.66 ± 0.70	2.96 ± 0.15	2.29 ± 0.32
Kidney	8.60 ± 1.88	7.77 ± 2.50	5.04 ± 0.41
Blood	7.20 ± 0.97	7.55 ± 0.47	6.71 ± 0.91

Each value represents the mean ± SD for three animals at each interval.

**Table 2:** Accumulation and contrast of ^123^I-BMIPP in a *P. aeruginosa* SR24 mouse thigh infection model by gamma counter.

Time after Injection (h)		Accumulation (%ID/g)	Contrast
1	Infected	6.3 ± 0.9	1.3
	Uninfected	4.8 ± 0.6	
2	Infected	6.7 ± 1.0	1.6
	Uninfected	4.3 ± 0.5	
4	Infected	5.6 ± 0.3	2.0
	Uninfected	2.8 ± 0.3	

Expressed as % injected dose (ID) per gram. Each value represents the mean ± SD for three mice at each interval.

**Table 3:** Accumulation and contrast of ^123^I-BMIPP in a *P. aeruginosa* SR24 mouse thigh infection model by SPECT imaging.

Time after Injection (h)		Accumulation (%ID)	Contrast
1	Infected	6.0 ± 1.4	1.5
	Uninfected	4.0 ± 0.5	
2	Infected	4.9 ± 0.6	1.7
	Uninfected	2.9 ± 0.3	
4	Infected	5.4 ± 0.4	2.3
	Uninfected	2.3 ± 0.3	

Expressed as % injected dose (ID). Each value represents the mean ± SD for three mice at each interval.

## Data Availability

All data are available in the article.
